# Effect of fungal chitosan on the morphology, biochemical, and genetic changes in selected genotypes of *Scutellaria barbata* D. Don under in vitro conditions

**DOI:** 10.1038/s41598-026-51097-7

**Published:** 2026-05-16

**Authors:** Justyna Lema-Rumińska, Magdalena Kulczyk-Skrzeszewska, Kamila R. Nowak, Jolanta Tyburska-Woś, Anna Frymark-Szymkowiak

**Affiliations:** Faculty of Biological Science, Department of Environmental Biology, Kazimierz Wielki University, 12 Ossolińskich Av., Bydgoszcz, PL-85-093 Poland

**Keywords:** Barbed skullcap, Anthocyanins, Carotenoids, Chlorophyll, Scutellarin, Secondary metabolites, SCoT marker, Free proline, Catalase, UPGMA, UPLC, Biochemistry, Biological techniques, Biotechnology, Microbiology, Plant sciences

## Abstract

*Scutellaria barbata* D. Don is an important plant for the phytopharmaceutical industry, and scutellarin is one of the key compounds produced in its shoots with confirmed anticancer properties. However, there is a lack of studies focused on obtaining phytochemically and genetically homogeneous, sterile plant material that is free from bacterial and fungal contamination and has a high scutellarin content. This study aimed to assess the effect of the biotic elicitor fungal chitosan, derived from *Aspergillus niger*, on the morphology of microcuttings, scutellarin content, concentrations of plant pigments (anthocyanins, carotenoids, chlorophyll a, and chlorophyll b), and selected oxidative stress biomarkers (free proline and catalase activity). Additionally, the study assessed genetic stability using Start Codon Targeted Polymorphism (SCoT) molecular markers in three genotypes of *S. barbata* (L5, L6, and L7). The addition of chitosan from *A. niger* to the medium influenced the morphological features, concentrations of anthocyanins, carotenoids, chlorophyll a, chlorophyll b, scutellarin, and free proline content, as well as the genetic stability of *S. barbata* microcuttings, with the effects depending on its concentration and genotype. However, catalase activity was not affected.

**Supplementary Information:**

The online version contains supplementary material available at 10.1038/s41598-026-51097-7.

## Introduction

*Scutellaria barbata* D. Don, commonly known as barbed skullcap, belongs to the Lamiaceae family. Under natural conditions, such as in Southeast Asia, it is a small perennial plant growing to about 50 cm in height; its genome consists of 26 chromosomes (2n)^1,2^. This plant has been used in traditional Chinese medicine (TCM) for hundreds of years, while in Western medicine, its medicinal properties are currently being intensively studied and confirmed^3,4^. Research by Wang et al.^1^ showed that this inconspicuous plant contains approximately 200 biologically active compounds. Among these, the most valuable secondary metabolites of *S. barbata* include scutellarin, a flavonoid whose anticancer activity has been confirmed in numerous scientific studies^5–8^. The mechanism of action of scutellarin involves modulation of signaling molecules such as STAT3, AKT, mTOR, and NF-κB, and suppression of key pathways in cancer cells, including JAK2/STAT3, PI3K/Akt/mTOR, Raf/MEK/ERK, HIPPO-YAP, and Wnt/β-catenin. These effects lead to inhibition of cancer cell growth and proliferation, angiogenesis, and induction of apoptosis^8,9^. In addition, this compound also possesses other valuable properties, including anti-inflammatory, antioxidant, antiplatelet, and antiphotoaging effects^1,10,11^. Therefore, it shows potential for use in treating various cardiovascular diseases and even Alzheimer’s disease^1,10,12,13^.

However, there is a lack of pure, phytochemically homogeneous, selected plant material with a high content of valuable phytochemicals available for industrial use. In vitro cultures offer a promising tool for the selection and multiplication of biomass from valuable plant genotypes (lines), as the material obtained through this method is phytochemically homogeneous and free from microbiological contamination and chemical residues of plant protection products (pesticides or herbicides). Despite this, the use of tissue cultures for research on secondary metabolites in *S. barbata* remains very limited. The first studies on seven selected lines of *S. barbata* were conducted by Lema-Rumińska et al.^14^. These studies enabled comparison of, among other factors, the content of valuable metabolites—including scutellarin—in new lines (genotypes) of microcuttings and plants grown under field conditions. However, under in vitro conditions, all tested genotypes showed significantly lower scutellarin content compared to those grown in the field, with in vivo content being 1.7 to 2.7 times higher depending on the genotype. Basal values ​​for scutellarin were the highest for the L5, L6, and L7 lines (8.43, 8.29, and 11.54 mg g^− 1^ in FW microcuttings, respectively). The current study continues this line of research by focusing on the selection of the most efficient genotypes regarding scutellarin content, combined with their elicitation in in vitro using chitosan (the choice of chitosan doses for selecting 50, 100, and 200 mg L⁻¹ is based on previously conducted preliminary studies; data not shown), as well as examining selected markers of oxidative stress (free proline and catalase) and assessing the genetic stability of the plants using molecular markers.

The increase in secondary metabolite content in plants can be achieved through the elicitation process in vitro^15^. Elicitors may be of biotic or abiotic origin^16^. One of the better-known biotic elicitors is chitosan, a biopolymer derived from chitin through deacetylation^17,18^. The most common source of chitosan is various marine invertebrates (e.g., crustaceans), but recent efforts have explored obtaining chitosan from selected fungal cell walls as well^19,20^. Due to its nontoxicity, chitosan is used across agriculture, horticulture, food, biomedical industries, and cosmetics^21–25^. The first systematic study on the effect of fungal elicitor chitosan was conducted by Agrawal et al.^26^. Their results contributed to a better understanding of defense mechanisms in *Oryza sativa* L. seedlings and the synthesis of antifungal phytoalexins, including the flavonoid sakuranetin and the diterpenoid lactone. There are also documented examples of using chitosan (mainly from invertebrates) to increase the content of valuable secondary metabolites in plants. For example, an increase in artemisinin content was observed in hairy roots of *Artemisia annua*^27^; flavonoids and phenolic compounds increased in callus cultures of *Ginkgo biloba*^28^; furanocoumarins increased^29^; phenolic compounds rose in cell suspensions of *Iberis amara*^30^; and an increase in wogononin and wogonoside (flavones) was reported in hairy roots of *Scutellaria lateriflora*^31^. The mechanism of chitosan’s action is not yet fully understood^17^. It is known to be based on the plant’s defense response to stress factors, such as fungal pathogens^26^. Plant cell membranes contain chitin-specific receptors that induce defense responses. When treated with a chitin-derived compound, plants activate their defense mechanisms because these compounds mimic molecules associated with chitin-containing organisms^17^.

In plant cells, under the influence of a stress factor, there is an overproduction of reactive oxygen species (ROS, including H₂O₂), against which the plant must defend itself to prevent cellular damage—such as lipid peroxidation in cell membranes, DNA damage, and other effects—that can lead to premature aging or even cell death. The first line of defense against the harmful effects of ROS includes, among others, low-molecular-weight amino acids like proline and plant pigments such as flavonoids and carotenoids. The second line of defense consists of specialized antioxidant enzymes, including catalase, which is one of the most effective enzymes in breaking down hydrogen peroxide into water and oxygen^32,33^. This natural defense mechanism of plants against stress is utilized in the elicitation process. As a result, the content of valuable compounds can be increased in the tissues subjected to elicitation^34^.

Genetic stability of plants in in vitro cultures may be disturbed, especially when plant growth regulators (PGRs) such as 2,4-D (2,4-dichlorophenoxyacetic acid) or BAP (6-benzylaminopurine) are added to the medium in large amounts. Additionally, the addition of other substances (external factors) to the medium that affect plant growth and development should be carefully controlled for their impact on genetic stability. To date, two molecular markers have been most commonly used to study genetic stability: RAPD (Random Amplified Polymorphic DNA) and ISSR (Inter Simple Sequence Repeat)^35–39^.

This study hypothesized that the chitosan from *Aspergillus niger* would significantly increase the biomass of microcuttings and the content of metabolites in *S. barbata* tissues through oxidative stress while maintaining genetic stability. The mechanistic pathways underlying defense/stress responses are not fully understood^26^. This study will fill the space by combining biochemical testing with morphological and molecular analysis to provide a better understanding of plant defense/stress mechanisms.

This study aimed to assess the effect of chitosan derived from *Aspergillus niger* on the morphology and content of scutellarin, anthocyanins, carotenoids, and chlorophylls a and b in microcuttings of three genotypes (lines) of *S. barbata* grown in vitro. In addition, the content of free proline and catalase—was examined. The obtained microcuttings were also analyzed using molecular markers of the SCoT (Start Codon Targeted Polymorphism) type to assess genetic stability.

## Materials and methods

### Plant material

The research material consisted of microcuttings of three genotypes (the most efficient lines) of *S. barbata* D. Don, selected for their scutellarin content, which were obtained in previous studies from seeds provided by Tadeusz Błaszczyk, MD, a doctor of Western and Chinese medicine practicing in Konstancin-Jeziorna, Poland^14^. All tested lines had an identical history regarding culture initiation, explant type, age of microcuttings, and number of passages. Microcuttings of each genotype were multiplied using the single-node shoot fragment method on modified MS medium^40^ without growth regulators. Passages lasted 4 weeks. The medium was modified by increasing the iron and calcium content by 50%. The pH of the medium (5.8) was adjusted before sterilization in a MICROJET (ML2-0127) microwave sterilizer (Enbio Technology Limited Liability Company, Gdańsk, Poland) at a sterilization temperature of 135 °C and an electromagnetic wave frequency of 2,450 MHz. After sterilization, the medium was poured in a laminar flow cabinet into sterile polypropylene “round” boxes with a capacity of 500 mL. Thirty in vitro cultures of 10 microcuttings each were obtained from each genotype (line). The research material was grown in a growth room at a temperature of 24 ± 2 °C, relative humidity of approximately 80%, and a 16-h photoperiod, with an average quantum irradiance of about 33.94 µmol m⁻² s⁻¹ from FLUORA G13 T8/36 W lamps (OSRAM, Germany).

### Effect of chitosan from *Aspergillus niger*

**Fig. 1:**
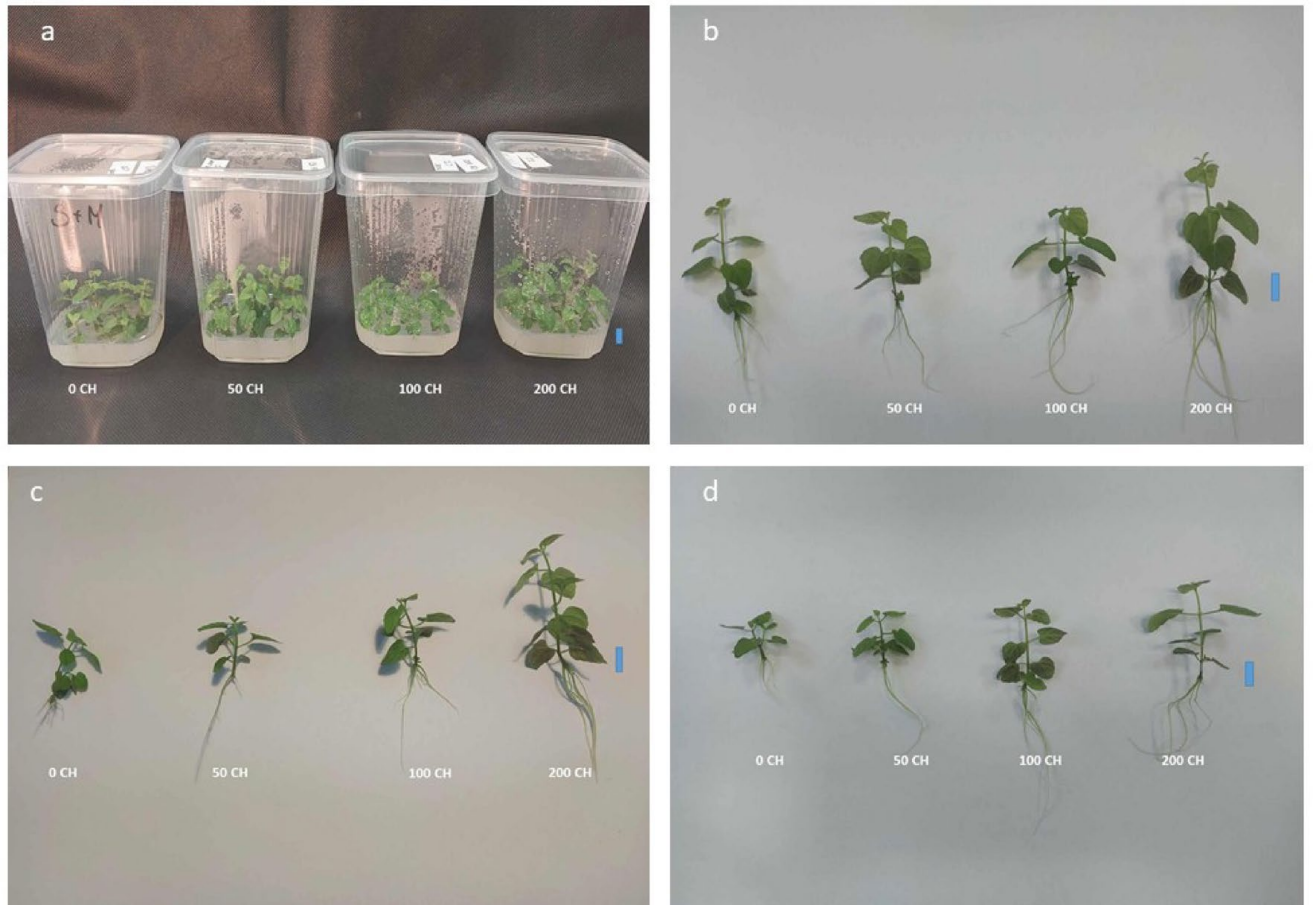
Morphological characteristics of the genotypes of *S. barbata* treated with chitosan from *A. niger* at different concentrations (0, 50, 100, and 200 mg L⁻¹; *bar* = 1 cm). (**a**) Microcuttings of the L7 line in the box. (**b**) Rooted microcuttings of L5 genotype. (**c**) Rooted microcuttings of L6 genotype. (**d**) Rooted microcuttings of L7 genotype.

The studies were conducted on modified MS medium^40^ without growth regulators (as described above), supplemented with different concentrations of chitosan extracted from *A. niger* (0 control, 50, 100, and 200 mg L⁻¹; 10–120 centipoise (cps), very low and low molecular weight, ≥ 85.0% degree of deacetylation (DDA); Chemat, Gdańsk, Poland). Single-node shoot fragments of three genotypes of *S. barbata* lines L5, L6, and L7 were used for the experiments (Fig. 1). For each genotype (line), 80 sterile polypropylene rectangular boxes were prepared, into which the medium was aseptically poured (20 boxes per chitosan concentration, totaling 80 boxes per line and 240 boxes for all lines combined). Each plant material for the study was collected from 10 randomly selected boxes with 10 microcuttings (for morphology, anthocyanins, carotenoids, chlorophyll a, and chlorophyll b, proline, catalase, and genetic studies, and scutellarin). Chitosan was added to the medium after pH adjustment and before sterilization. Each in vitro culture was inoculated with 10 single-node explants without leaf blades in a polar orientation. A total of 2,400 explants per genotype were inoculated. The in vitro cultures were maintained in a growth room under the same conditions described above. After 4 weeks, analyses were performed on the obtained microcuttings, including morphological assessment; total content of anthocyanins, carotenoids, chlorophyll a, and chlorophyll b; scutellarin; free proline; and catalase activity. Molecular analysis was also performed using the SCoT (Start Codon Targeted Polymorphism) marker.

### Morphological evaluation of genotypes (lines L5, L6, and L7) of *S. barbata* microcuttings obtained under the influence of chitosan from *A. niger*

For morphological evaluation, 24 microcuttings from each of the 3 genotypes (lines L5, L6, and L7), obtained from the shoot apices of microcuttings treated with different concentrations of chitosan (0, 50, 100, and 200 mg L⁻¹), were used, with 6 replicates for each treatment combination. The following morphological parameters of the microcuttings were evaluated: fresh weight, shoot length, number of nodes, number of leaves, and the length of the longest root.

### Biochemical studies of genotypes (lines L5, L6, and L7) of *S. barbata* microcuttings obtained under the influence of chitosan from *A. niger*

#### Spectrophotometric study of anthocyanins, carotenoids, chlorophyll a, and chlorophyll b in microcuttings

Plant pigments were extracted from fresh microcuttings of *S. barbata* genotypes L5, L6, and L7 (using 200 mg of fresh tissue for anthocyanin extraction and 100 mg for carotenoid extraction) following the procedure described by Lema-Rumińska and Zalewska^41^. The total contents of carotenoids and anthocyanins were calculated using coefficients from the equations of Wettstein^42^ and Harborne^43^, respectively. Chlorophyll a and b were extracted from fresh leaves using 100% acetone and 100 mg of fresh tissue samples, according to the method of Lichtenthaler and Buschmann^44^. Spectral analysis of the extracts was performed using a SPECTROstar *Nano* ultra-fast UV/Vis spectrophotometer (BMG Labtech GmbH, Ortenberg, Germany) in a 1 cm quartz cuvette at specific wavelengths (*λ*_max_): 530 nm for anthocyanins, 440 nm for carotenoids, and 645 and 662 nm for chlorophyll a and b, respectively. Pigment content was calculated in mg per gram of fresh weight, with the analysis performed in triplicate for each genotype and pigment type.

The algebraic method was used to quantify the concentration of total anthocyanins using the following formula:

$$\:{C}_{\mathrm{A}}=\frac{{A}_{530}}{h\times\:k}\left[\mathrm{g}\:{\mathrm{L}}^{-1}\right]$$

Where: *k* = 61.7 (extinction coefficient for 3-cyanidine glycoside) and *h* = 1 cm (layer thickness).

The concentration of carotenoids was calculated according to the formula:

$$\:{C}_{\mathrm{K}}=4.695\times\:{A}_{440}\:\left[\mathrm{m}\mathrm{g}\:{\mathrm{L}}^{-1}\right]$$

Chlorophyll a was calculated according to the formula:

$$\:{C}_{\mathrm{a}}=11.24\times\:{A}_{662}-\mathrm{2,04}\times\:{A}_{645}\:[\:{\upmu}\mathrm{g}\:\mathrm{m}\mathrm{l}$$]

Chlorophyll b was calculated according to the formula:

$$\:{C}_{\mathrm{b}}=20.13\times\:{A}_{645}-4.19\times\:{A}_{662}\:[{\upmu\:}\mathrm{g}\:\mathrm{m}\mathrm{l}$$]

#### Chromatographic analysis of scutellarin content in microcuttings using Ultra-High Performance Liquid Chromatography - Photodiode Array Detector (UPLC-PDA)

Air-dried samples were ground at room temperature (20 ± 2 °C) using a cutting mill. A dry weight (DW) of 0.100–0.120 g of rootless microcuttings from each line (L5, L6, and L7) and from media with different chitosan concentrations (0, 50, 100, and 200 mg L⁻¹) was taken for extraction. The plant material samples were subjected to the extraction process by adding 10 mL of a methanol/water solution (50:50 v/v) to the dry, ground sample of known mass. The sample was then placed in an ultrasonic cleaner for 30 min. Next, the sample was centrifuged at 6,000 rpm for 30 min, and the supernatant was transferred into a 50 mL volumetric flask. Extraction was performed twice for each medium and genotype combination. The combined extract was made up to volume with the methanol/water solution (50:50 v/v) in the volumetric flask and filtered through a 0.2 μm PVDF syringe filter.

The study was conducted using an ACQUITY H-Class UPLC PDA Waters liquid chromatograph equipped with an ACQUITY UPLC HSS T3 column (1.8 μm, 2.1 × 100 mm) employing gradient separation, with a peak retention time of 2.8 min. The column temperature was maintained at 40 °C, and the sample temperature at 10 °C. The injection volume was 1 µL. Mobile phase A consisted of 0.5% H₃PO₄ in water (water conductivity 0.09 µS cm⁻¹), and mobile phase B was 0.5% H₃PO₄ in acetonitrile (ACN). The detection wavelength was set at 325 nm, and the flow rate was 0.4 mL min⁻¹.

Scutellarin reference standard source (PhytoLab GmbH & Co., Vestenbergsgreuth, Germany), purity ≥ 95.00; calibration range: from 10,2 µg/ml to CAL5 to 51,0 µg/ml; linearity (R²) = 0,9996; LOD/LOQ - LOD = 0,05 µg/ml, LOQ = 0,143 µg/ml.

#### Spectrophotometric analysis of free proline and catalase activity

##### Free proline

The concentration of free proline in *S. barbata* microcuttings was prepared and measured following the method of Bates et al.^45^. Proline was extracted from 100 mg of fresh tissue [FW] using sulfosalicylic acid, and its reaction with ninhydrin was analyzed. For each sample, absorbance was measured at 520 nm using a CECIL spectrophotometer (CE 2011, 2000 series; Cecil Instruments, Cambridge, UK). A standard curve for proline was used for calibration. The analysis was performed in triplicate for each genotype. The results of free proline concentration were expressed as mg per gram of fresh weight (mg g⁻¹ FW).

##### Catalase activity

Fresh mass [FW] samples of *S. barbata* microcuttings (100 mg) were ground in an Eppendorf tube using a pestle in the presence of 50 mM phosphate buffer (pH 7.5) containing 1% (w/v) polyvinylpyrrolidone. The homogenate was centrifuged at 10,000×g at 4 °C for 20 min. The resulting supernatant was filtered through a 0.22 μm nylon syringe filter (Pureland, Stargard, Poland) and used for analysis. Analyses were performed in triplicate for each genotype. Catalase activity was determined spectrophotometrically according to the procedure developed by Aebi^46^, where the rate of H₂O₂ decomposition is monitored at 240 nm. The reaction mixture consisted of 50 µL of the original sample extract, 950 µL of phosphate buffer, and 500 µL of 30 mM H₂O₂ in buffer. Spectral analysis of the extracts was performed using a SPECTROstar Nano ultra-fast UV/Vis spectrophotometer (BMG Labtech GmbH, Ortenberg, Germany) in a 1 cm quartz cuvette. The standard of catalase came from bovine liver (Sigma-Aldrich, St. Louis, MO, USA), extraction coefficient E^1%^=36.6 (276 nm) ≥ 10,000 U mg⁻¹ protein, and the plant material came from in vitro cultures with constant moisture parameters and FW normalization.

### Studies on the genetic stability of *S. barbata* microcuttings treated with chitosan using SCoT molecular markers

The genetic stability of chitosan-treated microplants was assessed using molecular markers, specifically SCoT (Start Codon Targeted Polymorphism)^47^. Genomic DNA was isolated from 100 mg of fresh weight microplants of the L5, L6, and L7 genotypes (each sample/lane, in each treatment group, represents a pooled sample of 10 randomly selected microcuttings that are clones of a given line/genotype) following the protocol of the GeneMATRIX Plant & Fungi DNA Purification kit (EURx Ltd., Gdańsk, Poland). Total DNA concentration was determined using a BioPhotometer (Eppendorf, Warszawa, Poland). DNA analyses were performed twice using eight different SCoT primers (Genomed, Poland), with sequences provided in Table 1.

The reaction mixture contained: 1 µM of a single SCoT primer; 0.05 U·µL⁻¹ Taq DNA polymerase; 0.8 ng·µL⁻¹ template DNA; 1 mM dNTP solution mix; 2 mM MgCl₂ in reaction buffer; and sterile, nuclease-free double-distilled water, added to a final volume of 25 µL (2× PCR Master Mix Plus kit, A&A Biotechnology, Gdynia, Poland). The analysis was repeated twice. PCR reactions were performed using a BioRad C1000 Touch™ thermal cycler (Bio-Rad Laboratories, CA, USA) with the following program: initial denaturation at 94 °C for 4 min; followed by 45 cycles of denaturation at 94 °C for 1 min, annealing at 50 °C for 1 min, and DNA extension at 72 °C for 2 min. A final extension step was conducted at 72 °C for 4 min. PCR products were separated by electrophoresis on a 1.5% agarose gel and visualized under a UV transilluminator (GelDoc Go Photodocumentation System, Bio-Rad, Hercules, CA, USA) after staining with SimplySafe™ (EURx Ltd., Gdańsk, Poland). Molecular weights of the fragments were estimated using a 100–5,000 bp DNA molecular marker (GeneRuler™ Express DNA Ladder, Thermo Fisher Scientific, Waltham, MA, USA) and Bio-Rad Image Lab Software 6.1.0 (Bio-Rad Laboratories, Inc., CA, USA). The banding patterns were recorded as binary matrices (0–1), where “1” indicated the presence and “0” the absence of a specific fragment, followed by statistical analysis. For each primer tested, the numbers of monomorphic *loci*, polymorphic *loci* (present in the electrophoretic profiles of more than one individual), and specific/unique *loci* (present only in the profile of a single individual) were counted. Sample labeling on the gel is presented in Table 2. % Polymorphism was calculated according to the formula:

**Table 1:** SCoT primer sequences (5′→ 3′).

Primer SCoT	Sequences (5′→ 3′)
S4	CAACAATGGCTACCACCT
S12	ACGACATGGCGACCAACG
S13	ACGACATGGCGACCATCG
S25	ACCATGGCTACCACCGGG
S26	ACCATGGCTACCACCGTC
S27	ACCATGGCTACCACCGTG
S28	CCATGGCTACCACCGCCA
S33	CCATGGCTACCACCGCAG

$$\% ~Polymorphism = ~\frac{{~\left( {Polymorphic~bands + Specific~bands} \right)}}{{Total~bands}} \times 100$$

**Table 2:** Sample labeling in gel electrophoresis.

No. of sample	Concentration of chitosan [mg L^− 1^]	Designation of samples	Genotype (line)
1	0	0CH5	L5
2	50	50CH5	
3	100	100CH5	
4	200	200CH5	
5	0	0CH6	L6
6	50	50CH6	
7	100	100CH6	
8	200	200CH6	
9	0	0CH7	L7
10	50	50CH7	
11	100	100CH7	
12	200	200CH7	

### Statistical analysis

The Shapiro–Wilk test was used for testing normality, and Levene’s test for testing homogeneity of variances. A two-way ANOVA was used to examine the levels of significance (*p* ≤ 0.05) of the factors (genotype (line), chitosan concentration, and their interactions) on the studied morphological features and biochemical parameters (plant pigments and scutellarin content) of microcuttings (Table 3). Separate one–way ANOVA analyses were performed within genotypes. Mean values of morphological characters of the plants, as well as contents of plant pigments and scutellarin were separated using ANOVA (F-test). Levels of free proline and catalase activity were separated using Tukey’s honest significant difference test (HSD). Dendrograms were generated based on agglomerative hierarchical clustering (AHC) using the Unweighted Pair Group Method with Arithmetic Mean (UPGMA). Statistical analyses were performed with TIBCO Statistica™ 13.3 software (StatSoft Polska, Cracow, Poland).

## Results and discussion

Chitosan properties seem to be the most important factors that specifically stimulate plant responses. In this study, chitosan was deliberately selected from a pathogenic fungus to induce defense reactions. To our knowledge, this is the first study using *A. niger* to elicit a defense response in *S. barbata*. Additionally, the biopolymer was added directly to the medium before autoclaving to avoid salinity stress effects associated with preparing the chitosan solution.

**Table 3:** Results of a two-way ANOVA testing the influence of genotype (line) and concentration on morphological features, plant pigments content, and scutellarin content in microcuttings of *S. barbata.* Significant effects are bolded.

	Morphological features		Plant pigments		Scutellarin content	
	F	*p*	F	*p*	F	*p*
Genotype (Line)	1.71	0.124	1.20	0.321	**18.04**	**0.000**
Chitosan concentration	**2.64**	**0.007**	**3.23**	**0.001**	**12.22**	**0.000**
Genotype (Line) × chitosan concentration	1.53	0.084	1.20	0.274	**17.96**	**0.000**

The addition of fungal chitosan from *A. niger* to the medium had a significant effect on morphological features in *S. barbata* (Table 4). In particular, a dose of 200 mg L⁻¹ chitosan (cps 10–120, very low and low molecular weight (MW), ≥ 85.0% degree of deacetylation) showed a tendency of chitosan to increase the fresh weight, the number.

**Table 4:** Morphological characteristics of *S. barbata* genotypes (lines) treated with chitosan from *A. niger*. ^*^Data in columns (within a given genotype) are means ± SD, (*n* = 6). Different lowercase letters based on F-test at *p* ≤ 0.05 indicate significant differences in morphological features among chitosan concentrations in the medium in the individual lines of *S. barbata* by one–way ANOVA. of nodes and leaves, and the length of shoots and roots of microcuttings of *S. barbata* in most of the genotypes studied. Studies by Nge et al.^48^ also showed a positive effect of small amounts of chitosan on the growth and development of meristematic explants in the orchid *Dendrobium phalaenopsis*.

Genotype (line)	Concentration of chitosan [mg L^− 1^]	Microcutting fresh weight [g]	Number of nodes	Number of leaves	Shoot length [cm]	Longest root length [cm]
L5	0	0.096 ± 0.017a*	4.00 ± 0.63a	8.00 ± 1.26a	2.87 ± 0.44b	3.22 ± 1.24b
	50	0.073 ± 0.013b	4.33 ± 0.52a	8.67 ± 1.03a	3.27 ± 0.56b	3.55 ± 1.14b
	100	0.073 ± 0.016b	3.83 ± 0.41a	7.67 ± 0.82a	2.65 ± 0.18b	3.70 ± 0.81b
	200	0.090 ± 0.018a	4.00 ± 0.00a	8.00 ± 0.00a	3.45 ± 0.27a	4.85 ± 0.40a
L6	0	0.071 ± 0.015bc	4.00 ± 0.00b	8.00 ± 0.00b	2.98 ± 0.52b	3.90 ± 1.27b
	50	0.085 ± 0.016b	4.17 ± 0.41b	8.33 ± 0.82b	2.65 ± 0.21b	2.95 ± 0.40b
	100	0.074 ± 0.019c	3.66 ± 0.52b	7.33 ± 1.03b	2.80 ± 0.64b	3.27 ± 0.23b
	200	0.108 ± 0.022ab	4.50 ± 0.55a	9.00 ± 1.09a	4.40 ± 0.33a	5.40 ± 0.92a
L7	0	0.086 ± 0.012b	4.00 ± 0.63b	8.00 ± 1.26b	2.77 ± 0.39b	4.23 ± 0.56b
	50	0.087 ± 0.018b	4.17 ± 0.41b	8.33 ± 0.82b	2.85 ± 0.34b	5.22 ± 1.11b
	100	0.080 ± 0.014b	3.83 ± 0.41c	7.67 ± 0.82c	2.83 ± 0.44b	4.68 ± 0.70b
	200	0.115 ± 0.022a	4.50 ± 0.55a	9.00 ± 1.09ab	4.18 ± 0.33a	6.62 ± 1.82a

However, the authors emphasize that the type of chitosan and its molecular weight are critical factors. Specifically, a molecular weight of 100 kDa inhibited the growth and development of in vitro cultures in the tested orchid, whereas the optimal effect was observed with low molecular weight chitosan at 10 kDa, fungal chitosan at 20 ppm, or shrimp chitosan with a molecular weight of 1 kDa at 15–20 ppm^48^. Paris et al^49^. also proved that adding 250 mg L⁻¹ chitosan to the medium enhanced shoot formation in *Cattleya maxima*and improved the number of leaves, roots, and root length. Conversely, Mastuti et al^50^. found that the addition of chitosan to the medium had a less clear effect on shoot growth in seven genotypes of *Physalis*. Only one genotype (A2) showed significant shoot growth with the addition of 75 and 125 mg L⁻¹ chitosan. Carvalho et al^51^. reported a negative effect of chitosan on the growth of nodal segments in *Dysphania ambrosioides* (L.). Furthermore, studies by Krupa-Małkiewicz and Fornal^21^ showed that high molecular weight chitosan (970 kDa) at 15 ppm in MS medium under salt stress (100 mM NaCl) can alleviate the inhibitory effects of salinity on shoot and root growth in petunia ‘Prism White’. In contrast, low molecular weight chitosan (5 kDa) induces ROS and phytoalexin biosynthesis in petunia^21^.

In the production of secondary metabolites, increasing biomass production is highly important and can be applied to the tested genotypes of *S. barbata*. In our studies, we used chitosan from *A. niger* with very low and low molecular weights (10–120 cps), which probably may have induced plant defence/stress mechanisms and elicited the production of secondary metabolites. In our study, we found that high doses of chitosan (200 mg L⁻¹) caused a decrease in scutellarin concentration in the L5 and L7 lines but had no significant effect on scutellarin content in the L6 line (Table 5).

**Table 5:** Effect of chitosan from *A. niger* on scutellarin concentration in DW of *S. barbata* genotypes (lines L5, L6, and L7). ^*^Data in columns (within a given genotype) are means ± SD, (*n* = 3). Different lowercase letters based on F-test at *p* ≤ 0.05 indicate significant differences in scutellarin concentration among chitosan concentrations in the medium in the individual lines of *S. barbata* by one–way ANOVA.

Genotype (line)	Concentration of CH mg L^− 1^	Scutellarin [mg g^− 1^]
L5	0	16.369 ± 0.704a*
	50	12.645 ± 0.241c
	100	13.387 ± 0.368b
	200	11.753 ± 0.346d
L6	0	13.335 ± 0.997a
	50	12.698 ± 0.627ab
	100	14.761 ± 0.312a
	200	13.107 ± 0.407a
L7	0	13.161 ± 0.610a
	50	13.323 ± 0.275a
	100	13.597 ± 0.458a
	200	12.147 ± 0.322b

Previous studies by Lema-Rumińska et al^14^. reported that genotype L5 exhibited the highest scutellarin yield among the seven genotypes tested. Carvalho et al^51^. demonstrated a significant effect of chitosan on the synthesis of certain secondary metabolites; for example, a concentration of 200 mg L⁻¹ significantly increased the content of p-Cymene, Piperitone, and E-Ascaridole, while decreasing the concentrations of Terpinene and Z-Ascaridole in *D. ambrosioides*(L.). In our studies, we also observed a decrease in anthocyanin content in the L6 and L7 lines, as well as a reduction in carotenoids in the L6 line. Shah et al^52^. showed that the addition of chitosan (0.5–50.0 mg L⁻¹) stimulated the production of monolignols compared to the control medium in suspension cultures of *Silybum marianum* (L.), with the best results (increased silymarin content along with biomass) achieved at 5.0 mg L⁻¹ chitosan.

On the other hand, Pliankong et al^53^. reported that in cell cultures of *Catharanthus roseus* (L.) G. Don., the highest accumulation of vinblastine and vincristine (indole alkaloids) was obtained at 100 mg L⁻¹ chitosan with medium molecular weight.

A significant influence on the scutellarin concentration was found linked to the line, chitosan concentration, and both the line and chitosan concentration (Table 3). Our study indicates that an average dose of chitosan (100 mg L⁻¹) showed a general upward trend in scutellarin concentration across all tested genotypes (L5, L6, and L7) compared to the other chitosan concentrations. However, we were unable to statistically confirm the significance of this effect, suggesting that further research is required for a better understanding of the plant defense mechanisms in *S. barbata*. In the case of the L5 genotype, chitosan addition led to a decrease in scutellarin content even at the lowest tested concentration (50 mg L⁻¹). The L6 genotype did not show significant changes in the scutellarin level at the dose of 200 mg L⁻¹ compared to the dose of 100 mg L⁻¹ of chitosan (13.107 ± 0.407 to 14.761 ± 0.312 mg g⁻¹ DW of microcuttings, respectively), which may indicate good tolerance of the high dose of chitosan. Moreover, the average dose of chitosan (100 mg L⁻¹) also resulted in an upward trend in anthocyanin concentration in L5 and L6, and an increase in carotenoid content across all tested genotypes.

We also found significantly higher concentrations of chlorophyll a and chlorophyll b in the L5 genotype compared to the control and other chitosan treatments (Table 6). Rojas-Pirela et al^25^. noted the biostimulatory role of chitosan, which is comparable to that of plant growth-promoting bacteria (PGPB), and which also contributes to pigment biosynthesis.

It is interesting to note that the concentration of scutellarin observed in the current in vitro studies was 1.1 times higher for genotype L7 and up to 1.9 times higher for genotype L5, compared to the same genotypes in the previous study by Lema-Rumińska et al^14^.. These concentrations were nearly equivalent to the values obtained in vivo under field conditions, which is highly desirable. This outcome may be attributed to the type of light used in the growth room, a factor that warrants further research. In the present study, in vitro cultures were grown under Fluora G13 T8/36 W lamps, whereas Lema-Rumińska et al^14^. used daylight TLD 54/36 W lamps, both at similar light intensities. Carvalho et al^51^. showed a significant effect of light type on the synthesis of secondary metabolites in *D. ambrosioides* (L.). However, in the case of *S. barbata* D. Don, we did not specifically study the effect of light type; therefore, further studies are required to confirm the role of light type in the biosynthesis of scutellarin and other metabolites.

Our studies have shown a significant increase in free proline in the fresh weight of microcuttings across all tested *S. barbata* genotypes, which is probably related to the response to increasing chitosan concentrations in the medium.

**Fig. 2:**
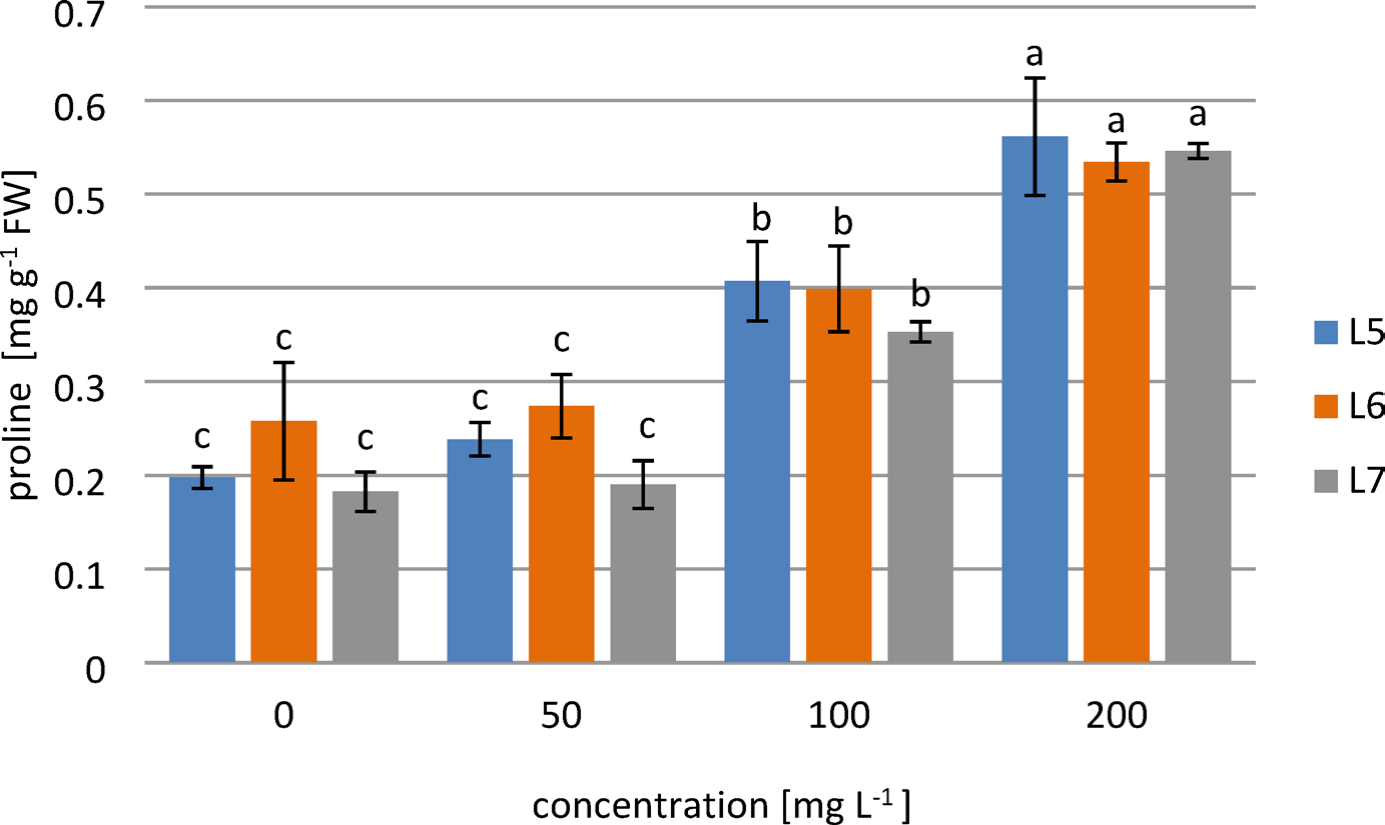
Proline accumulation in the fresh weight (FW) of microcuttings genotypes (lines L5, L6, and L7) of *S. barbata* treated with chitosan from *A. niger*. Data are means ± SD, (*n* = 3). Different lowercase letters based on Tukey’s test at *p* ≤ 0.05 indicate significant differences in proline accumulation among chitosan concentrations in the medium in the individual lines of *S. barbata* by one–way ANOVA.

In all tested *S. barbata* genotypes the significantly highest proline accumulation was detected in the fresh weight of microcuttings at the chitosan dose 200 mg L⁻¹ (L5–0.561 mg g⁻¹ FW, L6–0.534 mg g⁻¹ FW, L7–0.546 mg g⁻¹ FW) and the significantly lowest proline accumulation was found in the fresh weight of microcuttings without chitosan or at a low chitosan dose of 50 mg L⁻¹ (Fig. 2).

**Fig. 3:**
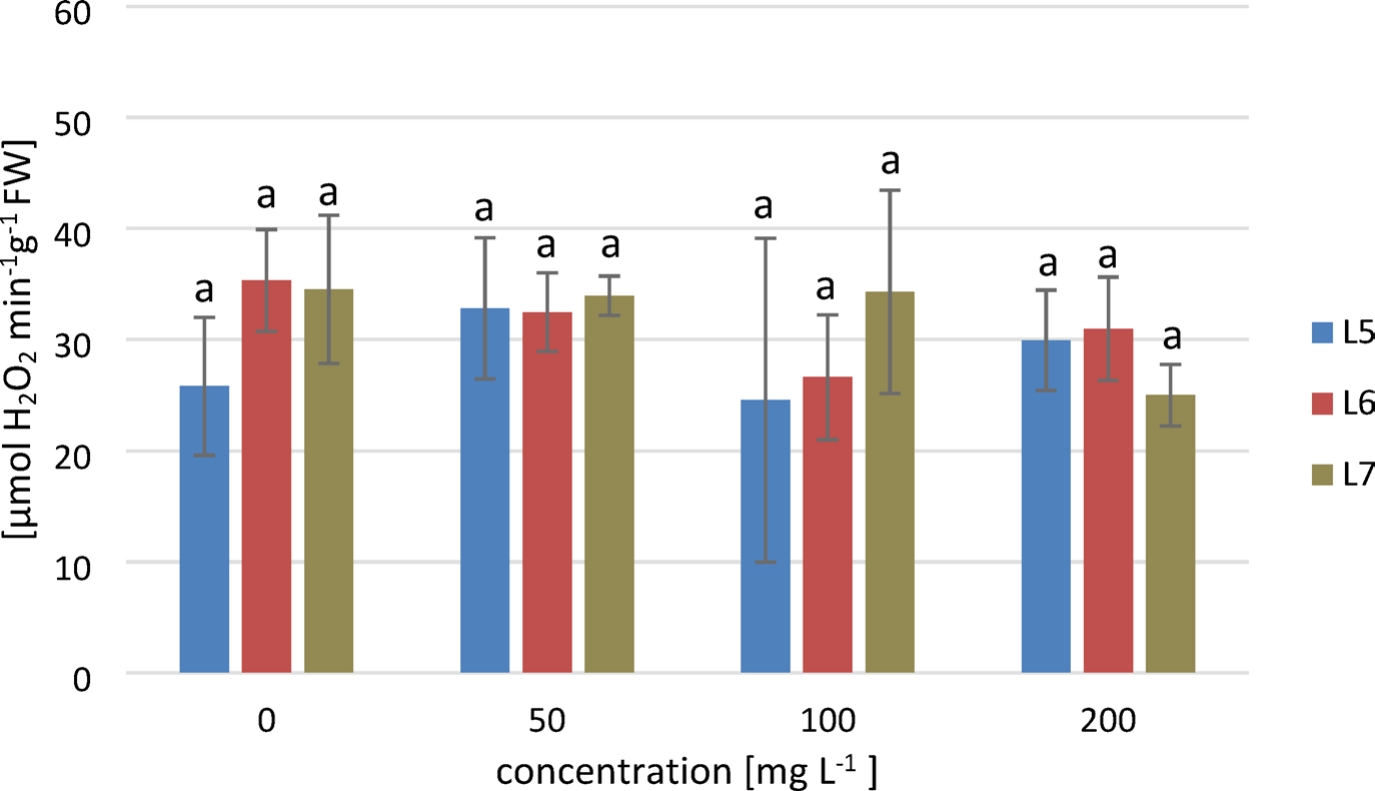
Catalase activity in the fresh weight (FW) of microcuttings genotypes (lines L5, L6, and L7) of *S. barbata* treated with chitozan from *A. niger*. Data are means ± SD, (*n* = 3). The same lowercase letters based on Tukey^’^s test at *p* ≤ 0.05 indicate no significant differences in catalase activity among chitosan concentrations in the medium in the individual lines of *S. barbata* by one–way ANOVA.

Proline regulates the expression of numerous genes and influences the growth and development of plants^54^. This probably may explain the increase in biomass and several morphological parameters observed in the tested *S. barbata *genotypes. Khanna-Chopra et al^55^. found that proline protects against stress by maintaining osmotic regulation and scavenging ROS, while stabilizing protein complexes in chloroplasts and the cytosol. The concentration of chitosan significantly influenced the analyzed plant pigment (Table 3). In our study, 100 mg L⁻¹ chitosan significantly increased the content of chlorophyll a and chlorophyll b in the fresh weight of microcuttings in the L5 genotype, and caused a noticeable upward trend in the other tested *S. barbata *lines. Khanna-Chopra et al^55^. demonstrated in tomato that proline increases chlorophyll a content. Proline acts as a first line of defense, together with carotenoids and flavonoids, protecting cells against ROS, including H₂O₂^32,56^. The addition of chitosan from *A. niger* to the medium did not affect catalase activity in *S. barbata*, which represents the second, enzymatic line of defense (Fig. 3).

**Table 6:** Effect of chitosan from *A. niger* on anthocyanin, carotenoid, chlorophyll a, and chlorophyll b concentrations in the fresh weight (FW) of microcuttings *S. barbata* genotypes (lines L5–L7). *Data in columns (within a given genotype) are means ± SD, (n=3). Different lowercase letters based on F-test at p ≤ 0.05 indicate significant differences in pigment concentration among chitosan concentrations in the medium in the individual lines of S. barbata by one–way ANOVA.

Genotype (Line)	Concentration of CH [mg L^−1^]	Anthocyanins	Carotenoids [mg g^−1^]	Chlorophyll a	Chlorophyll b
L5	0	0.449±0.131a*	0.667±0.084 a	1.095±0.100 b	0.503±0.064 b
	50	0.411±0.033a	0.654±0.054 a	1.099±0.104 b	0.499±0.031 b
	100	0.462±0.031a	0.790±0.050 a	1.331±0.061 a	0.578±0.028 a
	200	0.432±0.069a	0.682±0.144 a	1.152±0.226 ab	0.509±0.107 ab
L6	0	0.415±0.096a	0.667±0.020 a	1.143±0.022 a	0.373±0.096 a
	50	0.494±0.020a	0.593±0.036 b	0.983±0.057 b	0.436±0.027 b
	100	0.446±0.027a	0.723±0.076 a	1.210±0.081 a	0.524±0.045 a
	200	0.292±0.021b	0.585±0.067 b	1.008±0.110 ab	0.451±0.055 ab
L7	0	0.401±0.038a	0.646±0.060 a	1.095±0.104 a	0.543±0.088 a
	50	0.501±0.081a	0.680±0.108 a	1.119±0.218 a	0.516±0.070 a
	100	0.375±0.032ab	0.728±0.025 a	1.223±0.034 a	0.541±0.023 a
	200	0.325±0.033b	0.535±0.122 a	0.889±0.248 a	0.411±0.095 a

Catalase activity in all tested *S. barbata* genotypes did not differ significantly between different chitosan concentrations and was: 24.59–32.80 µmol H_2_O_2_ min^−1^g^− 1^ FW for L5 genotype, 26.61–35.32 µmol H_2_O_2_ min^−1^g^− 1^ FW for L6 genotype, 25.00–34.52 µmol H_2_O_2_ min^−1^g^− 1^ FW for L7 genotype (normalization of enzyme activity to FW of microcuttings). Although catalase is one of the most effective enzymes in breaking down hydrogen peroxide into water and oxygen^33^, its activity was not significantly higher at a dose of 200 mg L⁻¹ of chitosan in our study. This probably may indicate good tolerance of the dose. Studies investigating the effect of chitosan on oxidative enzymes, including catalase, have produced ambiguous results. Peykani and Sepehr^57^ showed that low concentrations of chitosan increased catalase activity, whereas high concentrations decreased it in maize and wheat seedlings under salinity stress. Conversely, Turk^58^ found that chitosan application under salinity stress induced catalase activity in maize seeds. Razavizadeh and Adabavazeh^59^ found that increasing chitosan concentration reduced catalase activity in *Dracocephalum kotschyi* (Lamiaceae) seedlings grown in vitro. Chitosan exerts a complex and variable effect on catalase activity in plants. This effect depends strongly on factors such as plant species, chitosan concentration, MW of chitosan, additional stress conditions, and application method. Therefore, the lack of significant differences in catalase activity in the present study does not necessarily indicate no effect, but rather suggests the need to optimize chitosan dosage and application conditions in future research.

The total number of products obtained using the SCoT marker for the tested *S. barbata* genotypes was 564, with band sizes ranging from 280 to 2,465 bp (Table 7).

**Table 7:** Characterization of molecular products obtained from tested *S. barbata* genotypes (L5, L6, L7) using the SCoT marker.

Primer	No. of products	Size range of products
S4	62	772–1873
S12	54	280–1906
S13	39	517–1653
S25	48	690–1026
S26	75	617–2465
S27	99	597–1352
S28	94	400–1172
S33	93	421–1849
Total	564	280–2465

**Fig. 4:**
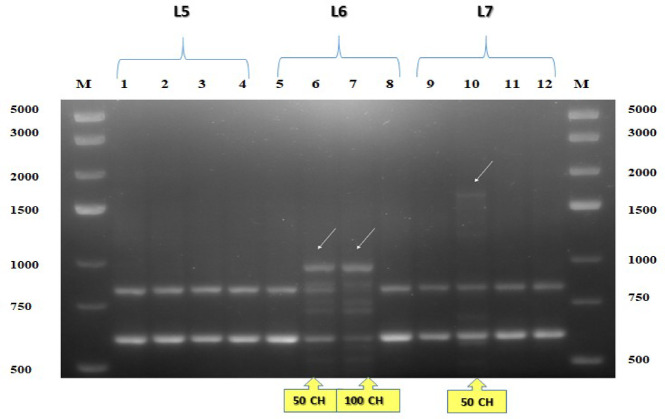
Effect of chitosan on the genetic stability of the tested genotypes L5, L6, and L7 of *S. barbata*. Example profile of products obtained using primer S13 of the SCoT marker (M = molecular standard; labeling in gel is given in Table 2. Each sample/lane in each treatment group represents a pooled sample of 10 randomly selected microcuttings that are clones of a given line/genotype; the gel was cropped; original gel can be found as Supplementary Fig. S1 online).

**Fig. 5:**
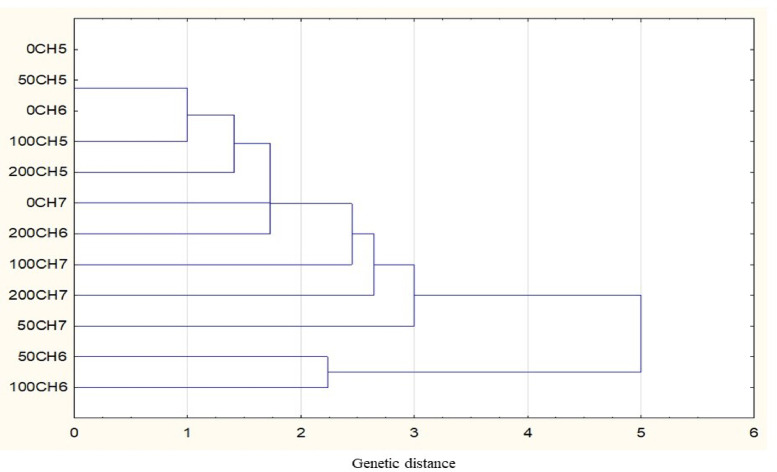
Dendrogram based on the estimation of real genetic distance and UPGMA clustering, presenting relationships between *S. barbata* genotypes L5, L6, and L7 treated or untreated (control) with chitosan using the SCoT marker (labeling is given in Table 2). Dendrogram was generated based on agglomerative hierarchical clustering (AHC) using the Unweighted Pair Group Method with Arithmetic Mean (UPGMA), also performed with TIBCO Statistica™ 13.3 software (StatSoft Polska, Cracow, Poland).

**Fig. 6:**
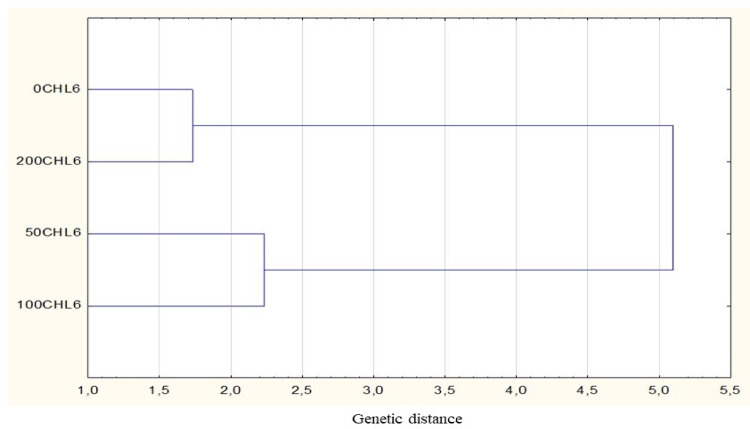
Sample dendrogram based on the estimation of real genetic distance and UPGMA clustering, presenting relationships within the *S. barbata* L6 genotype treated or untreated (control) with chitosan using the SCoT marker (labeling is given in Table 2). Dendrogram was generated based on agglomerative hierarchical clustering (AHC) using the Unweighted Pair Group Method with Arithmetic Mean (UPGMA), also performed with TIBCO Statistica™ 13.3 software (StatSoft Polska, Cracow, Poland).

Genotype L5 proved to be the most genetically stable among all tested lines (Fig. 4). Only primer S4 at the highest chitosan concentration (200 mg L⁻¹) showed polymorphism of 28.57% compared to the control. A slight decrease in polymorphism to control for this genotype was also observed at 100 and 200 mg L⁻¹ for primers S27 and S28, with reductions of 10.00% and 25.00%, respectively (Table 8). In previous studies by Lema-Rumińska et al^14^., using the ISSR marker system, genotype L5 formed a distinct cluster separate from all other tested lines, while the SCoT marker did not reveal any polymorphisms. In the current study, using SCoT markers, genotype L5 (0CH5: control) and those treated with various concentrations of chitosan form a subcluster exhibiting the smallest genetic distance (Fig. 5). Genotype L6 was identified as the most genetically unstable (Supplementary Table S1 online, Fig. 6). The majority (7) of SCoT primers used showed polymorphism levels ranging from 11.11% to 88.89%, depending on the chitosan concentration. The highest polymorphism (100%) was found in the L7 line with primer S12, although most SCoT primers did not detect polymorphisms for this genotype at different chitosan concentrations (Supplementary Table S2 online). Only a few studies in the literature have examined the effect of chitosan on the genetic stability of in vitro regenerated plants. Additionally, these studies report only a small degree of polymorphism in plants treated with chitosan^38,39,60^.

Studies conducted by Govindaraju and Arulselvi^38^ on *Coleus aromaticus *Benth (L.) showed no somaclonal variation in plants treated in vitro with chitosan (at concentrations ranging from 20 to 80 mg L⁻¹) in combination with the cytokinin BAP (6-benzylaminopurine), using RAPD markers. Similarly, Kandha et al^39^. found no significant deviations in the genetic stability of *Musa paradisica* plants regenerated in vitro under the influence of chitosan, as assessed by molecular markers such as ISSR and RAPD. Studies using RAPD markers conducted by Al-Mayahi^61^ on *Phoenix dactylifera *L. also did not reveal any genetic changes in plants treated with 15–20 mg L⁻¹ chitosan, either alone or in combination with thidiazuron. However, studies conducted by Samarfard et al^60^. on *Phalaenopsis gigantea* plants treated in vitro with chitosan and thidiazuron showed 27.3% polymorphisms after 16 weeks of culture, as detected by ISSR markers. In our studies, only chitosan was used, but at much higher concentrations in the medium, which resulted in a similar or higher level of somaclonal variability.

**Table 8:** Characterization of molecular products for *S. barbata* genotype L5 using the SCoT marker in response to varying concentrations of chitosan from *A. niger* (Mono –monomorphic bands, Poly – polymorphic bands, Spec – specific bands).

Primer	Concentration of chitosan [mg L^− 1^]	Mono	Poly	Spec	Total	% polymophism
S4	0 50 100 200	5 5 5 5	0 0 0 0	0 0 0 2	5 5 5 7	0.00 0.00 0.00 28.57
S12	0 50 100 200	3 3 3 3	0 0 0 0	0 0 0 0	3 3 3 3	0.00 0.00 0.00 0.00
S13	0 50 100 200	2 2 2 2	0 0 0 0	0 0 0 0	2 2 2 2	0.00 0.00 0.00 0.00
S25	0 50 100 200	4 4 4 4	0 0 0 0	0 0 0 0	4 4 4 4	0.00 0.00 0.00 0.00
S26	0 50 100 200	6 6 6 6	0 0 0 0	0 0 0 0	6 6 6 6	0.00 0.00 0.00 0.00
S27	0 50 100 200	9 9 9 9	1 1 0 0	0 0 0 0	10 10 9 9	10.00 10.00 0.00 0.00
S28	0 50 100 200	6 6 6 6	2 2 0 2	0 0 0 0	8 8 6 8	25.00 25.00 0.00 25.00
S33	0 50 100 200	7 7 7 7	0 0 0 0	0 0 0 0	7 7 7 7	0.00 0.00 0.00 0.00

## Conclusion

This is the first study examining the effect of chitosan from *A. niger* on morphological features, concentrations of anthocyanins, carotenoids, chlorophyll a, chlorophyll b, scutellarin, free proline content, and genetic stability of microcuttings in three selected *S. barbata* D. Don genotypes (L5, L6, L7). In this study, we observed a tendency for chitosan to increase the biomass of *S. barbata* microcuttings in vitro. High doses of chitosan (200 mg L⁻¹) significantly increased the fresh mass of microcuttings, the number of leaves and nodes, as well as the length of shoots and the longest roots across the genotypes studied. Metabolite content varied depending on both chitosan concentration and genotype. At high chitosan doses, the highest proline concentrations were observed in all tested genotypes, accompanied by decreases in scutellarin concentration (genotypes L5 and L7), anthocyanins (L6 and L7), and carotenoids (L6). At the medium chitosan dose (100 mg L⁻¹), chlorophyll a and chlorophyll b concentrations increased significantly in genotype L5, while an upward trend was noted for scutellarin across all genotypes, anthocyanins in L5 and L6, and carotenoids in L5, L6, and L7. However, chitosan did not affect catalase activity. Genotype L5 was the most genetically stable, with the highest level of polymorphism recorded at 200 mg L⁻¹ chitosan (28.57% for primer S4). The least genetically stable was genotype L6, which exhibited the highest number of polymorphisms, ranging from 11.11% to 88.89%. Further studies are necessary to better understand the influence of fungal chitosan on secondary metabolite production and oxidative stress in *S. barbata*.

## Supplementary Information

Below is the link to the electronic supplementary material.

Supplementary Material 1

Supplementary Material 2

## Data Availability

The data sets used and analysed during the current study are available from the corresponding author on reasonable request.

## Abbreviations

ACN: Acetonitrile
BAP: 6-benzylaminopurine
CH: Chitosan
Cps: Centipoise
DDA: Degree of deacetylation
2,4-D: 2,4-dichlorophenoxyacetic acid
DW: Dry weight
FW: Fresh weight
ISSR: Inter Simple Sequence Repeat
MS medium: Murashige and Skoog medium (1962)
M: Molecular standard
MW: Molecular weight
PGPB: Plant growth-promoting bacteria
PGRs: Plant growth regulators
RAPD: Random Amplified Polymorphic DNA
ROS: Reactive oxygen species
SCoT: Start Codon Targeted Polymorphism
TCM: Traditional Chinese medicine
UPLC-PDA: Ultra-High Performance Liquid - Photodiode Array Detector
