# Supplementary material for: Effect of fungal chitosan on the morphology, biochemical, and genetic changes in selected genotypes of Scutellaria barbata D. Don under in vitro conditions
Source: Sci Rep. 2026 May 16;16:22317. doi: 10.1038/s41598-026-51097-7 (PMC13376188; doi:10.1038/s41598-026-51097-7)
Supplement: Supplementary file 1 — Supplementary Material 1 [file 41598_2026_51097_MOESM1_ESM.docx]

| **Primer** | **Concentration of chitosan**  **[mg L^−1^]** | **Mono** | **Poly** | **Spec** | **Total** | **%**  **Polymophism** |
| --- | --- | --- | --- | --- | --- | --- |
| **S4** | 0  50  100  200 | 5  5  5  5 | 0  0  0  0 | 0  0  0  0 | 5  5  5  5 | 0.00  0.00  0.00  0.00 |
| **S12** | 0  50  100  200 | 3  3  3  3 | 0  6  6  0 | 0  0  0  1 | 3  9  9  4 | 0.00  66.70  66.70  25.00 |
| **S13** | 0  50  100  200 | 2  2  2  2 | 0  6  6  0 | 0  0  0  0 | 2  8  8  2 | 0.00  75.00  75.00  0.00 |
| **S25** | 0  50  100  200 | 2  2  2  2 | 2  2  2  2 | 0  0  0  0 | 4  4  4  4 | 50.00  50.00  50.00  50.00 |
| **S26** | 0  50  100  200 | 6  6  6  6 | 0  1  1  0 | 0  0  1  0 | 6  7  8  6 | 0.00  14.28  25.00  0.00 |
| **S27** | 0  50  100  200 | 2  2  2  2 | 7  4  2  7 | 1  0  0  0 | 9  6  4  9 | 88.89  66.67  50.00  77.78 |
| **S28** | 0  50  100  200 | 8  8  8  8 | 0  0  0  0 | 0  0  1  0 | 8  8  9  8 | 0.00  0.00  11.11  0.00 |
| **S33** | 0  50  100  200 | 6  6  6  6 | 1  2  1  1 | 0  0  0  1 | 7  8  7  8 | 14.28  25.00  14.28  25.00 |

**Supplementary Table S1** Characterization of molecular products for *S. barbata* genotype L6 using the SCoT marker in response to varying concentrations of chitosan from *A. niger* (Mono –Monomorphic bands, Poly – Polymorphic bands, Spec – Specific bands).

| **Primer** | **Concentration of chitosan**  **[mg L^−1^]** | **Mono** | **Poly** | **Spec** | **Total** | **%**  **Polymophism** |
| --- | --- | --- | --- | --- | --- | --- |
| **S4** | 0  50  100  200 | 5  5  5  5 | 0  0  0  0 | 0  0  0  0 | 5  5  5  5 | 0.00  0.00  0.00  0.00 |
| **S12** | 0  50  100  200 | 0  0  0  0 | 4  4  1  2 | 1  1  0  3 | 5  5  1  5 | 100.00  100.00  100.00  100.00 |
| **S13** | 0  50  100  200 | 2  2  2  2 | 0  0  0  0 | 0  3  0  0 | 2  5  2  2 | 0.00  60.00  0.00  0.00 |
| **S25** | 0  50  100  200 | 4  4  4  4 | 0  0  0  0 | 0  0  0  0 | 4  4  4  4 | 0.00  0.00  0.00  0.00 |
| **S26** | 0  50  100  200 | 6  6  6  6 | 0  0  0  0 | 0  0  0  0 | 6  6  6  6 | 0.00  0.00  0.00  0.00 |
| **S27** | 0  50  100  200 | 5  5  5  5 | 4  1  4  3 | 0  0  0  0 | 9  6  9  8 | 44.44  16.67  44.44  37.50 |
| **S28** | 0  50  100  200 | 8  8  8  8 | 0  0  0  0 | 0  0  0  0 | 8  8  8  8 | 0.00  0.00  0.00  0.00 |
| **S33** | 0  50  100  200 | 8  8  8  8 | 0  1  1  1 | 0  0  0  1 | 8  9  9  10 | 0.00  11.11  11.11  20.00 |

**Supplementary Table S2** Characterization of molecular products for *S. barbata* genotype L7 using the SCoT marker in response to varying concentrations of chitosan from *A. niger* (Mono –Monomorphic bands, Poly – Polymorphic bands, Spec – Specific bands).
