# Supplementary material for: Effect of fungal chitosan on the morphology, biochemical, and genetic changes in selected genotypes of Scutellaria barbata D. Don under in vitro conditions
Source: Sci Rep. 2026 May 16;16:22317. doi: 10.1038/s41598-026-51097-7 (PMC13376188; doi:10.1038/s41598-026-51097-7)

**Supplementary Figure S1.** Amplification results obtained using the primer S13 of the SCoT marker. The gel image corresponds to the Figure 4 in the main manuscript.


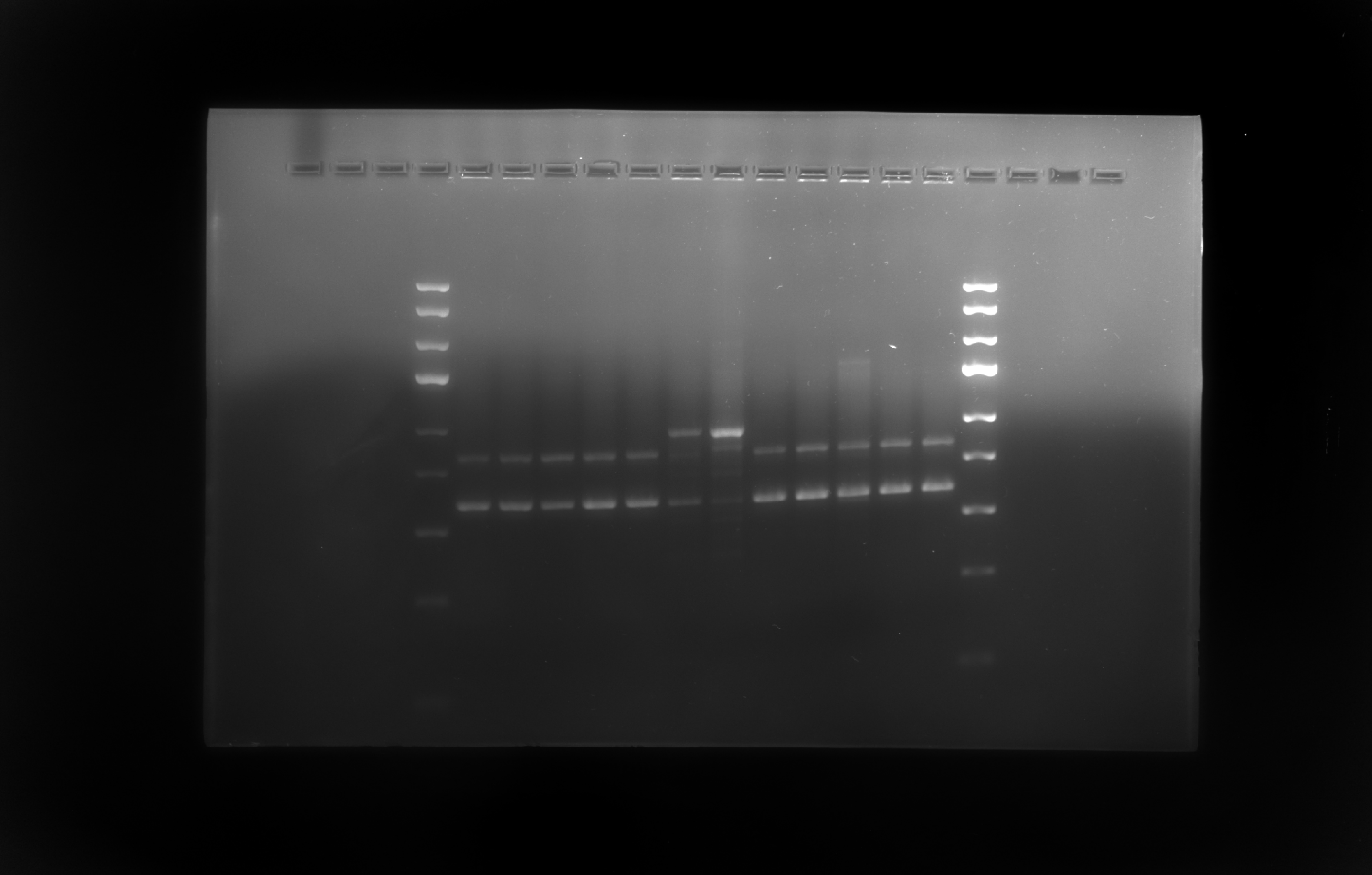


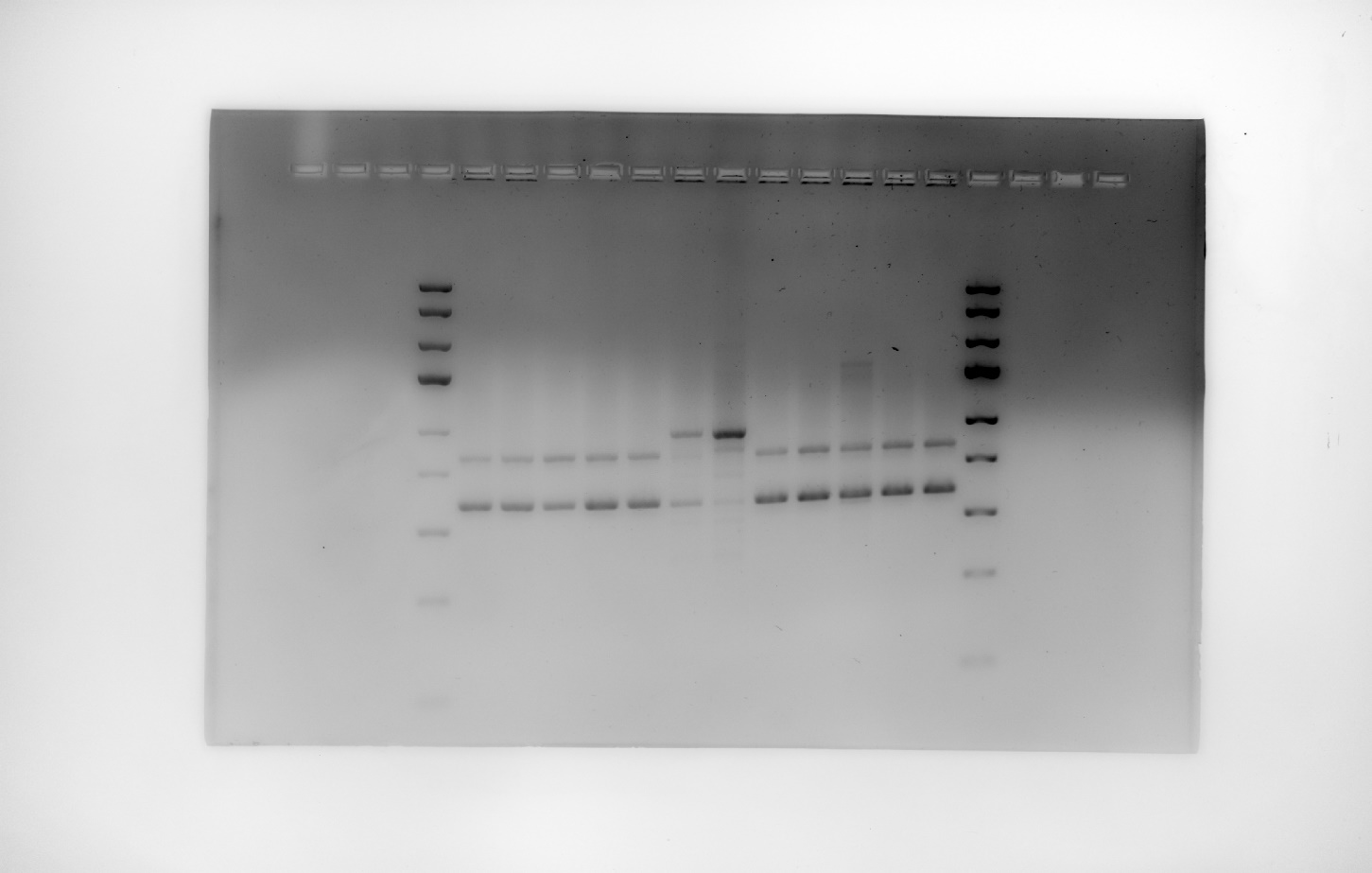

Supplement: Supplementary file 2 — Supplementary Material 2 [file 41598_2026_51097_MOESM2_ESM.docx]
